# Complete genome sequence of the *Streptomyces* bacteriophage Amabiko

**DOI:** 10.1128/mra.00182-24

**Published:** 2024-04-23

**Authors:** Mark Milhaven, Heba A. Bakry, Anuvi Batra, Amanda M. Bermingham, Gloria Grama, Jacob Kebe, Shawn S. Martinez, Rishika V. Mudunuri, Megan R. Nelson, Evie T. Nguyen, Mia M. Peterson, Alexis Pruitt, Kristan Tran, Akarshi Brar, Gabriella Cerna, Elaine Chaffee, Steven M. Caruso, Susanne P. Pfeifer

**Affiliations:** 1 School of Life Sciences, Arizona State University, Tempe, Arizona, USA; 2 Department of Biological Sciences, University of Maryland, Baltimore, Maryland, USA; 3 School of Mathematical and Statistical Sciences, Arizona State University, Tempe, Arizona, USA; 4 School of Molecular Sciences, Arizona State University, Tempe, Arizona, USA; 5 Department of Psychology, Arizona State University, Tempe, Arizona, USA; 6 School of Human Evolution and Social Change, Arizona State University, Tempe, Arizona, USA; 7 Center for Evolution and Medicine, Arizona State University, Tempe, Arizona, USA; Queens College, Flushing, New York, USA

**Keywords:** bacteriophage assembly

## Abstract

Amabiko is a lytic subcluster BE2 bacteriophage that infects *Streptomyces scabiei*—a bacterium causing common scab in potatoes. Its 131,414 bp genome has a GC content of 49.5% and contains 245 putative protein-coding genes, 45 tRNAs, and one tmRNA. Amabiko is closely related to *Streptomyces* bacteriophage MindFlayer (gene content similarity: 86.5%).

## ANNOUNCEMENT

Here, we report the genome of Amabiko, a subcluster BE2 bacteriophage that infects *Streptomyces scabiei* RL-34—a Gram-positive, soil-borne, bacterial pathogen that causes common scab in potatoes (1).

**Fig 1:**
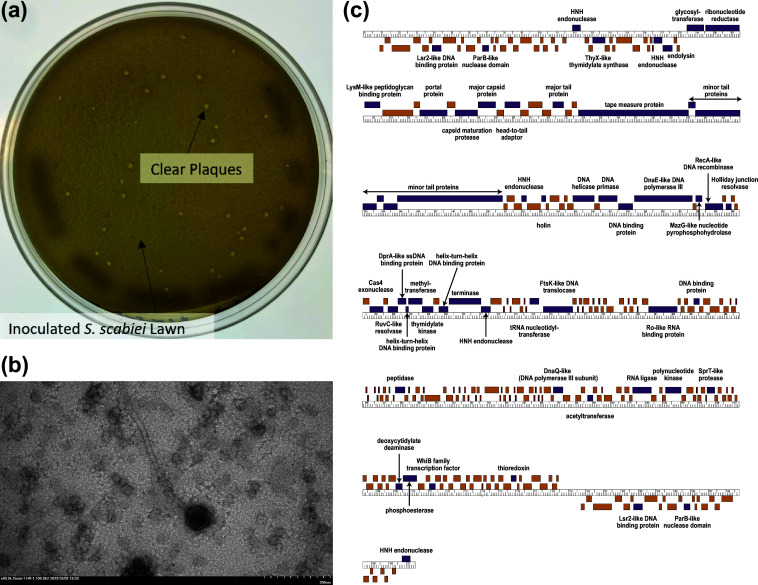
Characteristics of the *Streptomyces* bacteriophage Amabiko. (a) Amabiko forms ~2 mm clear, symmetrical, circular plaques. (b) Negative-stain (1% uranyl acetate) transmission electron microscopy image of Amabiko. Amabiko exhibits a siphoviral morphology, with a head length and width of 77 nm and an uncontracted tail length of 333 nm (*n* = 1; scale bar is located in the corner of the image). (c) Amabiko's complete genome sequence, containing 245 putative genes. The ruler indicates the length of the genome in kilobase pairs. Boxes represent individual genes transcribed in the forward and reverse direction (above and below the ruler, respectively), with purple boxes indicating genes that could be assigned a putative function and orange boxes indicating genes of unknown function. tRNAs and the tmRNA are shown as red boxes.

Amabiko was isolated from a soil sample collected in Baltimore, MD, USA, near a stream on the University of Maryland campus (39.25623 N, 76.71286 W). Following the SEA-PHAGES *Phage Discovery Guide* (2), the soil sample was suspended in phage buffer (10 mM Tris [pH 7.5], 10 mM MgSO_4_, 68 mM NaCl, 1 mM CaCl_2_), shaken for ~1 hour, centrifuged for 5 minutes, and filter sterilized (3). A plaque assay was performed by plating aliquots of the filtrate on cultures of *S. scabiei*. Specifically, *S. scabiei* cultures were inoculated with the filtrate for 10 minutes, added to tryptic soy top agar (BD), and overlaid on nutrient agar (BD Difco) supplemented with 10 mM MgCl_2_, 8 mM Ca(NO_3_)_2_, and 0.5% glucose (NA+). After 24 hours at 30°C, ~2 mm clear, symmetrical, circular plaques appeared, containing bacteriophage Amabiko (Fig. 1A). Amabiko was purified using three rounds of plaque picking combined with serial dilution. Negative-staining electron microscopy demonstrated that Amabiko has a siphoviral morphology, with a head length and width of 77 nm and an uncontracted tail length of 333 nm (Fig. 1B). A host range analysis demonstrated that Amabiko is able to infect closely related hosts (Table 1).

**TABLE 1:** Host range analysis of Amabiko, indicating the effectiveness of plating (EOP) of each strain, or the relative titer of the bacteriophage on a given cell line relative to the titer of the isolation host *S. scabiei* RL-34 (*n* = 1)

Strain	Effectiveness of plating (EOP)
*S. azureus* SC 2364, NRRL B–2655	none
*S. coelicolor* subsp. coelicolor, NRRL B–2812	none
*S. diastatochromogenes* IFO 3337, NRRL ISP–5449	KFW^*a*^
*S. griseus* subsp. *griseus*, NRRL B–2682	6.00
*S. mirabilis*, NRRL B–2400	4.67
*S. scabiei* RL-34, ATCC 49173	1.00

^*a*^ KFW = “killing from without” (a phenomena that occurs when bacteriophages cause bacterial lysis without infection).

Amabiko's DNA was isolated from freshly harvested plate lysate and extracted using the Wizard genomic DNA purification kit (Promega), and a sequencing library was prepared using the Illumina TruSeq DNA Nano Library preparation kit. The library was sequenced on an Illumina NovaSeq 6000, yielding 242,285 single-end 150 bp reads (238-fold coverage). Read quality was checked using FastQC v0.12.1 (https://www.bioinformatics.babraham.ac.uk/projects/fastqc/), and no adapter sequences or low quality bases were detected. Reads were assembled using the “*De Novo* Assembly” option in the CLC Genomics Workbench v.6.5.1, resulting in a 131,414 bp contig with a GC content of 49.5%. Accuracy, completeness, and genomic termini were verified using the consensus sequence editor Consed v29.0 (4).

Following the SEA-PHAGES *Bioinformatics Guide* (5), Amabiko was identified as a *Streptomyces* subcluster BE2 bacteriophage. A total of 245 putative genes were identified using DNA Master v5.23.6 (http://cobamide2.bio.pitt.edu), GLIMMER v3.02 (6), GeneMark v2.5 (7), and Starterator v1.0.1 (https://seaphages.org/software/#Starterator). Out of the 245 putative genes, 57 genes (23.4%) could be assigned a putative function based on evidence from bacteriophages available in BLASTp v2.13.0 (8) (using information from the Actinobacteriophage database and the non-redundant protein database), HHpred v2.08 (9) [using information from the Protein Data Bank (PDB)_mmCIF70_24_Oct, Pfam-A_v36, Uniprot-SwissProt-viral70_3_Nov_2021, and NCBI_Conserved_Domains(CD)_v3.19], and Phamerator (http://phamerator.org) (Fig. 1C). Nine genes (3.7%) could be identified as transmembrane proteins using SOSUI v1.11 (10) and TMHMM v2.0 (11). Additionally, 45 tRNAs and one tmRNA were identified using Aragorn v1.2.41 (12) and tRNAscan-SE v2.0 (13). All software was run with default settings.

The Gene Content tool on phagesdb was used to calculate the gene content similarity between Amabiko and other subcluster BE2 bacteriophages, demonstrating that Amabiko is closely related to MindFlayer (GenBank accession number: MW291014) (gene content similarity: 86.5%).

## Data Availability

The whole-genome sequencing data are available through NCBI Sequence Read Archive (BioProject accession number PRJNA488469; run number SRR27983391). The annotated genome assembly is available through NCBI GenBank under accession number PP358748.

## References

[1] Ismail S, Jiang B, Nasimi Z, Inam-Ul-Haq M, Yamamoto N, Danso Ofori A, Khan N, Arshad M, Abbas K, Zheng A. 2020. Investigation of Streptomyces scabies causing potato scab by various detection techniques, its pathogenicity and determination of host-disease resistance in potato germplasm. Pathogens 9:760. doi:10.3390/pathogens9090760 32957549 PMC7559370

[2] Poxleitner M, Pope W, Jacobs-Sera D, Sivanathan V, Hatfull G. 2018. SEA-PHAGES phage discovery guide. Howard Hughes Medical Institute, Chevy Chase, MD. Available from: https://seaphagesphagediscoveryguide.helpdocsonline.com/home

[3] Sarkis GJ, Hatfull GF. 1998. Mycobacteriophages, p 145–174. In In mycobacteria protocols. Humana Press, New Jersey.

[4] Gordon D, Abajian C, Green P. 1998. Consed: a graphical tool for sequence finishing. Genome Res 8:195–202. doi:10.1101/gr.8.3.195 9521923

[5] Pope WH, Jacobs-Sera D, Russell DA, Cresawn SG, Hatfull GF. 2017. Howard Hughes medical Institute. In SEA-PHAGES bioinformatics guide. Chevy Chase, MD.

[6] Delcher AL, Harmon D, Kasif S, White O, Salzberg SL. 1999. Improved microbial gene identification with GLIMMER. Nucleic Acids Res 27:4636–4641. doi:10.1093/nar/27.23.4636 10556321 PMC148753

[7] Lukashin AV, Borodovsky M. 1998. GeneMark.hmm: new solutions for gene finding. Nucleic Acids Res 26:1107–1115. doi:10.1093/nar/26.4.1107 9461475 PMC147337

[8] Altschul SF, Gish W, Miller W, Myers EW, Lipman DJ. 1990. Basic local alignment search tool. J Mol Biol 215:403–410. doi:10.1016/S0022-2836(05)80360-2 2231712

[9] Söding J, Biegert A, Lupas AN. 2005. The Hhpred interactive server for protein homology detection and structure prediction. Nucleic Acids Res 33:W244–8. doi:10.1093/nar/gki408 15980461 PMC1160169

[10] Hirokawa T, Boon-Chieng S, Mitaku S. 1998. SOSUI: classification and secondary structure prediction system for membrane proteins. Bioinformatics 14:378–379. doi:10.1093/bioinformatics/14.4.378 9632836

[11] Krogh A, Larsson B, von Heijne G, Sonnhammer EL. 2001. Predicting transmembrane protein topology with a hidden Markov model: application to complete genomes. J Mol Biol 305:567–580. doi:10.1006/jmbi.2000.4315 11152613

[12] Laslett D, Canback B. 2004. ARAGORN, a program to detect tRNA genes and tmRNA genes in nucleotide sequences. Nucleic Acids Res 32:11–16. doi:10.1093/nar/gkh152 14704338 PMC373265

[13] Lowe TM, Eddy SR. 1997. tRNAscan-SE: a program for improved detection of transfer RNA genes in genomic sequence. Nucleic Acids Res 25:955–964. doi:10.1093/nar/25.5.955 9023104 PMC146525

